# Pathogenesis and host response to a novel Tacaribe virus isolate in experimentally-infected Jamaican fruit bats

**DOI:** 10.64898/2026.06.12.731631

**Published:** 2026-06-12

**Authors:** Phillida A Charley, Luke Namesnik, Paul S. Soma, Ashley B. Reers, Clara Reasoner, Shijun Zhan, Bradly Burke, Liliana M Dávalos, Jan Felix Drexler, Allison C. Vilander, Rushika Perera, Hannah K Frank, Corey L. Campbell, Tony Schountz

## Abstract

Tacaribe virus (TCRV) was the first arenavirus discovered in the New World and was isolated from *Artibeus* bats in Trinidad and Tobago in the 1950s. One isolate, TRVL-11573, remains but it was passaged by intracranial inoculation of newborn mice 22 times that likely changed its biology. This isolate has been extensively used for arenavirus research, including our previous work that showed it can cause fatal neurological disease in Jamaican fruit bats ( *Artibeus jamaicensis* ). Another divergent TCRV, DOM2014, was recently identified from a Jamaican fruit bat captured in the Dominican Republic that contained TCRV genome. A kidney fragment homogenate from this bat was inoculated into Jamaican fruit bats and all became infected with signs of mild liver disease. Experimental challenge of Jamaican fruit bats with DOM2014 led to nonfatal infection that persisted through the end of the study on day 21 and with contact transmission to naive bats. Histopathology, immunohistochemistry and serum chemistry confirmed infection and mild liver disease, but none of the bats produced neutralizing antibodies. B cell receptor transcripts suggested limited somatic hypermutation that could explain the lack of detectable neutralizing antibodies. Transcriptome profiling of livers and spleens showed signatures of a typical innate antiviral response; however, evidence of adaptive immune suppression was also present. Similarly, liver transcriptome analysis showed signatures of an expected innate antiviral response and metabolic dysfunction. The isolation of TCRV-DOM2014 provides a relevant model for the study of a bat reservoir host, and which may challenge the extensive work previously conducted with TRVL-11573.

**IMPORTANCE:**

Several New World arenaviruses cause disease in humans and many are BSL-4 agents. TCRV strain TRVL-11573 has been used since the 1950s to study arenavirus biology; however, because it was intracranially passaged in newborn mice and Vero cells, it likely accumulated mutations that changed its biology. This assertion has been reinforced in recent years with discovery of divergent TCRV sequences in wild *Artibeus* bats that are substantially different than TRVL-11573, thus the prototype strain is unlikely to represent wildtype TCRV. The isolation of a new TCRV strain that has retained its genome fidelity allows a better understanding of TCRV’s biology and pathogenic potential. The availability of pathogen-free Jamaican fruit bats and cell lines that are permissive for TCRV-DOM2014 will also help retain the biological features of the virus. Collectively, this is among the most tractable bat reservoir host models developed and provides a system for dissection of how bats host viruses. Moreover, it may be a suitable model for the study of therapeutics and vaccines for New World arenaviruses.

## Full Text Availability

The license terms selected by the author(s) for this preprint version do not permit archiving in PMC. The full text is available from the preprint server.

